# Capillary Circulation

**Published:** 1858-06

**Authors:** D. L. M’Gugin

**Affiliations:** Professor Physiology and Pathology, Iowa University


### IOWA MEDICAL JOURNAL.

J. C. HUGHES, } EDITORS. { WELLS R. MARSH.

VOL. IV. KEOKUK, IOWA, APRIL, MAY & JUNE, NO. 4.

### ORIGINAL COMMUNICATIONS.

### CAPILLARY CIRCULATION.

BY D. L. M'GUGIN, A. M., M. D.

(Professor Physiology and Pathology, Iowa University.)

The circulation of the blood in the capillary vessels has heretofore been the theme of much speculation, and a subject of close investigation.

Different theories have been advanced, some of which were highly plausible, and in all this there was but an approximation to the truth. The different opinions formed and avowed were so diverse in character that it was very plain there was no satisfactory settlement in the medical mind on the subject, and showed too that the results of the researches had not established a uniform or general sentiment as to the *modus* of its performance. The vital, the chemical, and physical, with certain combinations of these, were presented by their several advocates, as the true interpretations of the mode, and the manner, of the act performed. Albeit there has been some progress in this as with other questions heretofore and still doubtful, and yet the end had not been attained, nor in our opinion was the truth revealed. The position in which the subject was left, the explanations of the laws governing it, the agent or agents whose vital activity contributed to its production, were unsatisfactory interpretations of the phenomena and their causes. In this perplexity we were compelled to acknowledge that a cloud hung darkly over this subject as well as others in medical science.

The goal reached is a "capillary power" which is too abstract and ambiguous without an explanation of the agents and instruments in its production. When we observe certain acts or processes, the mind is incited to an inquiry into the cause or causes, which, if discovered, the phenomena are better comprehended and understood. If these agents remain obscure and unrevealed, then we fall back to a position as nearly as possible approximating to truth, which is to acknowledge that certain phenomena manifest themselves, but the instruments by which they are performed, are unknown. This appeared to be the unfortunate position of this physiological act, for as phenomena were manifest, an agent was presumed but undiscovered, and as there must be a certain force in its performance, it was termed *power*. Since then there has been an apathy on the subject for other questions withdrew from it the attention of research, although no one more important in a physiological, pathological, or practical point of view.

It was, therefore, both natural and reasonable that enquiries should be instituted into the phenomena first, then into the probable cause in their production. But the experimental enquiries and researches have been too limited and unsatisfactory, and therefore the attempt at investigation was discouraging at the outset.

We had so often observed, during the prevalence of cholera asphyxia, the great loss, indeed, the almost entire destruction of the circulation in the capillaries, that we naturally associated with that loss, some mal-condition of the blood resulting in these morbid manifestations. The blood might be negatively bad, and its mal-condition be the result of deficiency of its normal elements; or it may prove to be positively so by the presence of an additional and noxious element in its perversion or destruction.

The lividity present pointed to an excess of carbon, by which the oxygen might be overwhelmed and therefore prove inoperative. The condition of the respiration showed that the normal physiological changes had not taken place in the lungs, for no warmth is felt by the breath expired.

The condition of the blood in the arteries and the unorganizable state of the blood when drawn from a person laboring under cholera, showed that the fibrin had lost its vitality. As fibrin is but oxygenized albumen, the results of the examination into the circulating fluid after death, as found in the arteries, plainly showed a deficiency of oxygen, and an increase of carbonic acid. There was also a great diminution of the proportion of salts, and hence, admitting that the lungs performed their functions, æration or vitalization could not well take place because these vehicles for the transmission of oxygen did not exist. The inference was that both conditions obtained, namely, that there was an excess of carbonic acid, and a diminution of oxygen. We then attempted the application of these facts to the subject matter of an enquiry into the circulation in the capillaries and asked the question, how far do these conditions go toward the interruption and destruction of this special function?

Before we proceed to answer this question we will refer the reader to certain experiments which go far toward establishing the fact that, the presence of the normal proportion of oxygen in the blood, is absolutely necessary to its circulation. In the asphyxia of cholera it has been observed upon trial, that the inhalation of oxygen did result in partially awakening the circulation into greater activity, and that there was a partial restoration of temperature. The effect, though temporary, was nevertheless very manifest, and we have no good reason for attributing the partial revival of these vital demonstrations to any other cause than to the introduction of a new supply of oxygen. The excess of carbon, doubtless, tended to embarrass and stultify the circulation, conjoined to the increased spissitude because of the loss of its normal proportion of water.

Then again, when the blood is defective in the amount of its solid constituents and therefore more fluid, it is also defective in its oxygenized material. It is an admitted physiological fact, that blood in this condition will not circulate as freely as when of normal spissitude. This is a reversal of the commonly observed law of physics, for a sponge will absorb thin fluids more rapidly and readily than those of more consistence. Water will flow through textures, or fine cylindrical tubes more promptly and freely than oleaginous or syrupy mixtures.

We must account for the passage of blood in these fine vessels, upon some other grounds and for other reasons than upon its consistence simply. Its flow must depend upon some power beyond it; to some chemical condition of the structure in which these vessels freely ramify, and to which they are bearing nutrition. The simple condition of spissitude will not answer the requirement. There must be present some impressible agent upon which exterior and more potent influences will be excited in order to invite it onward.

The before mentioned experiment of introducing oxygen into the blood by inhalation, fully establishes in its results two important facts; first, that a preponderance of carbon in the blood is attended with an interruption in its circulation; and second, that an increase of oxygen will revive more or less its motion in the capillaries. As a legitimate consequence of the renewal of the blood's movement, there is an increase in the temperature. The deduction from these facts would be that in the blood contained within the capillaries and flowing onward, the oxygen is the *primum mobile* in the circulating fluid itself. It being the chief agent in combustion, the increase of temperature which follows is the result of its action and agency and establishes its presence.

Although for obvious reasons, these manifestations of returning vitality have not proved to be permanent there were nevertheless, evidences sufficiently strong to show that to its introduction and presence, the phenomena were owing.

The fact that in the excitement of a part, there is an increase of the circulation with an exalted temperature, is another important feature in the capillary function. Local excitements, when independent of the heart, show an exalted capillary movement to that part, yet, at the same time, the momentum does not proceed from the heart but is invited by the local excitement.

The capillary vessels are the vehicles for the transmission of nutrition for the repairment of the waste in the tissues, and in proportion to the free distribution of these vessels and the activity in which nutrition is performed, there is a corresponding vigorousness in the movement of the blood. Where is this nutritive material expended and disposed? In the tissues in which they ramify and are diffused. The capillary circulation is then the *nutritive or reparative* circulation, and its peculiarities are doubtless adapted to the most efficient performance of that act. The condition of the circulation will be an index to the state of nutrition, for if the circulation be limited in quantity, or interrupted in its flow, nutrition is thereby modified or suspended. That the capillary circulation is in some way connected with nutrition, and that the last mentioned physiological act is performed through the operation of the former, seems to be now generally admitted.

In his work on "Human Physiology," Carpenter (when referring to this process in the lower animals and plants,) uses the following language: "No physiological fact is more clearly proved than the existence, in the lower classes of animals, as well as in plants, of some power, independent of the *vis a tergo* by which the circulating fluid is made to move through their vessels. This power seems to *originate* in themselves and to be closely connected with the state of nutrition and secreting processes, since anything which stimulates them to increased energy, accelerates the circulation; whilst any check to them occasions a corresponding stagnation. It may be convenient to designate this moter force by the name of *capillary power;* it being clearly understood, however, that no *mechanical* propulsion is thence implied."

After presenting the different phenomena as manifest in the circulation within the capillaries, he proceeds (on page 494) to say that "If the phenomena which have been here brought together be considered as establishing the existence, in all classes of beings possessing a circulating apparatus, of a "capillary power" which affords a necessary condition for the movement of the nutritious fluid, through those parts in which it comes in more immediate contact with the solids, the question still remains open what is the nature of that power?"

Then again, when discussing the question of an independent contractility on the part of the capillary vessels, he denies the existence of such an agent in the movement of the blood. "Hence the notion of any mechanical assistance, afforded by the action of the walls of the capillaries to the movement of the blood through them, must be altogether dismissed. There is experimental evidence, however, that the movement of the blood may be affected by any agency which alters the chemico-vital relations between the blood and the tissues which it permeates."

Again (on page 495,) "That alterations in the chemical state of the blood (involving of course important changes in its vital properties,) are capable of exercising a most important effect on the capillary circulation, is shown, not merely by the stagnation of the pulmonary circulation in asphyxia, but by the curious fact ascertained by Dr. Reid that the blood when imperfectly arterialized, is retarded in the *systemic* capillaries, causing an increased pressure upon the walls of the arteries."

The capillary circulation then is in "*some mysterious way*" connected with nutrition. The mechanical agency is disavowed. The power of the heart, if it exercises any influence whatever, is believed to be of second importance, certainly not the chief power. The larger arteries perform no part and the doctrine of ordinary capillary attraction is entertained only by a few. If confined to these vessels and the fluid they contain, how is it performed when the vessels give no evidence of alternate distension and contraction as in the arteries? They serve only to give direction to the course of the fluid and at the same time confine it within its limits. We must look in part for the cause in the blood itself, and this too while discharging its legitimate duties in the repairment of the tissues, which are all the while wasting away.

In this investigation we recognise the existence of a law of diffusion, and this takes place through their affinitive relations. This obtains between carbon, hydrogen and oxygen; in a particular manner between the former and the latter. They are in close relation in all forms of organization, and very many of the phenomena connected with it, are dependent upon, and are the result of their connexion. The higher orders of organization demand the oxygen, while the vegetable products exchange it for carbon. To the former it is a life element, while to the latter it is destructive in its tendency and requires the oxygen to nullify its effects. Even while this is being performed, important results to health and life follow.

The death of tissues is attended with carbon while the living acknowledge the presence and preponderance of oxygen. In the wasted or decayed tissues, carbon is the chief product, while the new supply holds a preponderance of oxygen. In the former, a vacuum, in a certain sense, is produced, or we should say the rather that a negative state obtains, while in the blood there is a plenary condition with regard to oxygen. Now comes the play of affinities. This negative condition must be overcome, the loss supplied, and the oxygen is called upon to answer this demand.

Hence the activity of the oxygenized blood in these vessels, because attracted by the carbon of the effete matters in the tissues, and hence too the agency of nutrition in the performance of this act. This then, in my humble belief, is the "capillary power" of Carpenter, this the chemico-vital act which results in the establishment and continuance of this special circulation.

When nutrition ceases through the destruction of a part, as in sphacelus, in which the capillaries are exempt from destruction, the circulation ceases also. The motion of the blood is not arrested because the vessels themselves are incompetent, but because extensive disorganization and the consequent loss of vitality, have destroyed the progress of nutritive assimilation in the lost tissues surrounding.

Touching the affinity which exists between certain elements of the blood and tissues, Williams, in his Principles, (page 121) on the subject of changes in the blood, remarks that, "the conversion of the venous into arterial blood, comprises the absorbtion of oxygen, the removal of some carbonic acid and water, a slight increase of fibrin, and possibly other changes. Each of these elements of the process is probably concerned in giving to arterial blood its fitness for its functions; the absorbed oxygen by its affinity for the hydrogen and carbon of the blood and textures, aiding in those processes by which these are renovated in function as well as in structure, superfluous fat, and other combustible matters consumed, and heat is evolved, etc. etc.

These changes occurring in the lungs are not the only changes which the blood undergoes, for as oxygen is carried along the current to the capillaries it subserves an important purpose in the repairment of tissues. The operation here is similar to that performed in the lungs, and the presence of the oxygen is not only demanded, but is acknowledged. In the lungs, the blood is endowed and fitted for ulterior purposes, the chief of which is for the maintenance and repairment of tissues.

The heart, however, in propelling the blood thus qualified, sends it only to the capillaries, whose action is doubtless independent of the heart. In this supposition we are borne out by the fact that in the embryo-fœtus the capillary system is first organized, and the circulation is active in them before the heart is formed. Then in the acardiac fœtus this circulation is performed without the heart. Then again, the other instance, to which we have already referred; where the surrounding tissues are destroyed, but where the capillaries remain intact and patulous, yet the heart fails to send streams of blood through them.

If priority of organization would give color to the claim of being ranked as of superior importance, then the capillary system would be entitled to it. Although this may not be the case in *every* instance, still it is but just to give to it an *independency* in its action. If it were deemed a first essential in the progress of organization and development during uterine life, there is no good reason to suppose that it is any the less important after birth, or that the function may not be performed *per se*, as well as the heart itself, through certain vital agencies and influences peculiar to itself.

The power exerted should be termed "*interstitial power*" rather than "capillary power." Kirkes denies to the capillaries a contractile or propulsing force on the part of their structures. He says: "It may be stated, with tolerable certainty, that the capillaries themselves possess no such power, and that the influence which they seem to exercise on the movement of their contained blood, is referable chiefly to the action of the small arteries, and in part, perhaps, to the results of the relation which exists between the tissues outside, and the blood within the capillaries."

Here is an admission that some relation does exist between the blood within the vessel and the tissues outside in propelling the blood. On the next page, however, he expresses a doubt upon the subject. But, as he makes the capillaries passive in the performance of this function, he must give to the heart and the arteries the sole influence. This, we have shown, to be highly improbable. If they were the chief contributors to the performance of the act, the *mode* and *manner* would be the same. The heart dilates and contracts, and the arteries which come under its influence do so also synchronously with it. The arteries are under its control, and they obey the law or mode of action which it observes itself. There is no rebounding, and therefore no pulsatory motion in the capillaries, but the movement of the contained fluid differs in that the current is constant and uniform. Differing then in the *manner* of the movement, it is but reasonable to suppose it must look to a different agent or influence also.

Draper, however, approaches more nearly the true solution of the capillary problem, when he says: "The relation between the interspaces of the capillaries and the blood thus introduced into them continues the current. The particular mode in which this relation is manifested, differs in different parts. The oxydizing arterial blood has a high affinity for those portions that have become wasted; it affects their disintegration and then its affinity is lost."

In this paragraph he does not designate the particular constituent for which it holds an affinity. In this work from which we make the extract, and which it will be remembered, was published in 1856, four years after we avowed the theory we now advocate; he has failed to explain the particular operation of these affinities or name any other active agent, save that of the oxygenized arterial blood, and that it disintegrates the effete particles. We maintain then

1st. That the heart has no immediate agency in the capillary circulation.

2d. That the small arteries contribute no further to the act than to send the blood to the capillaries.

3d. That this special circulation is the result of the affinity of the carbon, of the wasted tissues and the oxygen of the blood.

4th. That it is a chemico-vital act.

This doctrine we have maintained during the last five years, as is well known to our colleagues and the several classes of the College of the above period. A number of letters in our possession at this time will confirm this statement.

Very recently, however, we have seen an abstract in the "*Virginia Medical Journal*," taken from an article on the subject of the "capillary circulation," by Austin Flint, M. D., Buffalo, N. Y., in July last. We have not yet seen the article in full, but upon perusal of the abstract our views would appear to be somewhat similar. We addressed that gentleman a letter respectfully requesting a copy of his article, but up to this moment have received no reply. On this topic there has been exchange of opinion on the subject between us.

We shall bide our time until we see the article alluded to, but should we find our long cherished opinions sustained and confirmed by one so deservedly eminent and whose opinions are entitled to so much consideration, we will feel highly gratified and encouraged.

