# Errata

**Published:** 1858-06

**Authors:** 


**Errata.**

On page 250, 9th line from top, read *propulsive* for propulsing. Page 252, 5th line from top should be read "there has been *no* exchange," &c.

